# Interactions of multidomain pro-apoptotic and anti-apoptotic proteins in cancer cell death

**DOI:** 10.18632/oncotarget.28031

**Published:** 2021-08-03

**Authors:** Alexander Chota, Blassan P. George, Heidi Abrahamse

**Affiliations:** ^1^Laser Research Centre, Faculty of Health Sciences, University of Johannesburg, Doornfontein 2028, South Africa

**Keywords:** apoptosis, caspases, cancer, Bcl-2 family proteins, p53

## Abstract

Cancer is a global public health concern that is characterized by the uncontrolled growth of tumor cells. It is regarded as the subsequent cause of death after cardiovascular disease. The most common types of cancer include breast, colorectal, lung, and prostate. The risk factors attributed to the development of common types of cancer are tobacco smoking, excessive alcohol consumption, dietary factors, ultraviolet radiation (UV), and lack of physical activities. Two major cellular apoptotic pathways targeted in cancer therapies are intrinsic and extrinsic. These two pathways are regulated by different types of proteins, the multidomain pro-apoptotic proteins (Bak, Bax, and Bok), BH3-only pro-apoptotic proteins (Bid, Bim, Bad, Noxa, and Puma), and the anti-apoptotic proteins (Mcl-1, Bfl-1, Bcl-X_L_, Bcl-2, Bcl-w, and Bcl-B). Other significant molecules/factors that are known to execute cellular apoptotic pathways include bioactive compounds, and reactive oxygen species (ROS). Proteolytic caspases are known to play a vital role in the initiation of apoptotic activities in cancerous cells. Based on their functions, they are categorized into initiators and executioners. Nanotechnology has produced novel outcomes in modern medicine. The green synthesis of nanoparticles has demonstrated prospective improvements in cancer therapies in combination with the existing therapies including photodynamic therapy. This review aims at highlighting the association between pro-apoptotic and anti-apoptotic proteins, and their significance in cancer therapy.

## INTRODUCTION

Globally, cancer remains the main public health concern. Cancer is denoted by the continued growth and proliferation of cancerous cells [1, 2]. According to the GLOBOCAN estimates of 2020 reported by the International Agency for Research on cancer, the incidence rate of cancer was estimated at 19.3 million cases excluding 18.1 million non-melanoma skin cancers, and 10 million cancer-related deaths excluding 9.9 million non-melanoma skin cancers occurred in 2020 [3]. There are different types of cancers, the most prevalent types are breast, lung, prostate, and colorectal cancers [4]. In Sub-Saharan Africa, a high incidence of breast, prostate, and cervical cancers has been reported [5, 6].

Many risk factors attribute to the development of cancer. They include tobacco smoking, alcohol consumption, diet, ultraviolet radiation, infections, and lack physical activities [7]. Assessment of these risk factors is a cardinal component of cancer monitoring and evaluation as it gives a comprehensive overview of disease progression [8]. Innovative therapeutic approaches focusing on current perspectives have been developed from medicinal plants exhibiting anticancer activities. When compared to conventional treatment options such as chemotherapy and radiotherapy, phytochemicals and natural products are known to have fewer side effects [9, 10]. Targeted therapy is one of the promising cancer treatment options that target specific sites such as tumor intracellular organelles and specific proteins involved in tumor development. In photodynamic therapy, oxidative stress on organelles including the mitochondria, and endoplasmic reticulum (ER) is well known to induce the generation of Reactive Oxygen Species (ROS) [9].

Apoptosis is a form of cellular death that follows a programmed mechanism, which regulates various metabolic processes such as homeostasis. There are two major types of apoptotic pathways through which tumor cells undergo cell death, the intrinsic and extrinsic pathways [11]. The intrinsic pathway is activated by various intracellular activities e.g., mitochondrial oxidative stress whereas the extrinsic apoptotic pathway is induced by extrinsic factors which interact with tumor necrosis factor receptors (TNFR) [12]. Manipulation of apoptosis in various medical conditions and diseases has become one of the vital road maps employed by researchers to improve therapeutic modalities. For cancer cells to avoid apoptosis, they need to have the suppressing potential of toxic ROS, which may arise either from induced metabolic or environmental activities [13]. There are different forms of ROS with the ability to promote apoptosis in both normal and cancer cells such as hydrogen peroxide (H_2_O_2_). Therefore, it is critical for researchers involved in the development of better therapeutic options to consider optimizing the levels of ROS to be generated during therapy as it prevents proliferation and cancer recurrence [14]. In addition, manipulation of apoptosis in the resistant framework can significantly improve the treatment of diseases such as cancers.

The regulation of apoptosis is dependent on the expression of several anti-apoptotic proteins such as Mcl-1, Bfl-1, Bcl-X_L_, Bcl-2, Bcl-w, and Bcl-B [15]. These anti-apoptotic proteins prevent apoptosis through inhibition or inactivation of apoptotic proteins (Bak, Bax, and Bok). Bak, Bax, and Bok are apoptotic proteins whose function is to promote apoptosis. These three types of apoptotic proteins perform the same function, they are responsible for the permeabilization of the mitochondrial outer membrane which results in the release of cytochrome c [16]. Furthermore, there are five major BH3-only pro-apoptotic proteins initiators include Bid, Bim, Bad, Noxa, and Puma. The function of these pro-apoptotic proteins is to promote apoptosis via the inhibition of anti-apoptotic factors [17].

## PHOTODYNAMIC THERAPY

Photodynamic therapy (PDT) also known as light therapy is a localized non-surgical therapeutic modality used in the treatment of various diseases including different types of cancer. There are different types of light used in PDT, among them are the blue, and red light [18]. In this type of therapy, chemical agents known as photosensitizers are administered in affected body sites and later exposed to specific wavelengths. Once exposed to a specific light source for a specified period, the photosensitizer gets excited, and begins to interact with molecular oxygen, and initiates the production of cytotoxic ROS [19]. The consequences of having high production of ROS in a cell results in oxidative stress to the vital cellular organelles such as the mitochondria, endoplasmic reticulum, and peroxisomes. Nucleic acids (i.e DNA and RNA) are another vital cellular macromolecules that get damage through ROS-induced oxidative stress. However, damage to these significant cellular components may lead to cell dysfunction, and eventually cell death. Apoptosis is an example of a cell death mechanism that is demonstrated in PDT of cancer. With the invention of nanotechnology, photosensitizing nanoparticles have been synthesised from plants and used as potent plant-derived anticancer photosensitizing compounds to enhance the treatment efficacies. In contrast to synthetic anticancer compounds used in photodynamic therapy, green synthesised nanocompounds produce eco-friendly, and cost-effective anticancer agents. Silver nanoparticles are one of the best examples of such compounds used in PDT applications [20].

## TARGETED CANCER THERAPY

Cancer cells are dependent on dysfunctional apoptotic pathways for their survival and existence. There is a variety of DNA repair mechanisms that have the capacity, and potential to repair dysfunctional tumor apoptotic pathways and these include cell cycle checkpoints in G_1_ and G_2_/M-phase. In cancer therapy, the development of a targeted approach that induces cell apoptosis without interfering with the normal physiology of noncancerous cells is the future therapeutic goal in cancer treatments. It also aims at halting or interfering with well-defined molecules that induce tumor cell proliferation [21]. However, many phytochemicals have been reported to exhibit targeted apoptotic activities in many cancers as shown in Table 1. Some of these compounds are aloe-emodin from *Rheum ultimatum,* actinodaphnine from *Annona hypoglauca,* genistein found in soybeans, curcumin from *curcuma longa*, quercetin found in citrus fruits, green vegetables, onion, apple, and green tea, resveratrol found in grapes, baicalein from *Scutellaria baicalensis,* licochalcone A from *Glycyrrhiza glabra,* etc.

**Table 1:** Major anticancer compounds from plants and cell death mechanisms

Source/plant origin^a^	Compounds^b^	Chemical structure^c^	Mechanism of action^d^	Cancer type^e^	Reference
*Rheum plamatum*	Aloe-emodin	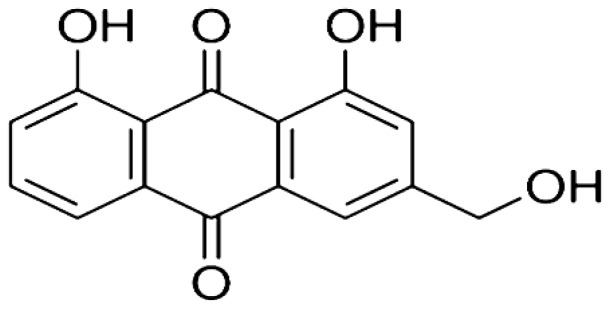	Induces the generation of ROS which causes oxidative damage to the mitochondrial outer membrane. Upregulates the level of p53 and p21 proteins.	Skin cancer	[22–24]
*Annona hypoglauca*	Actinodaphnine	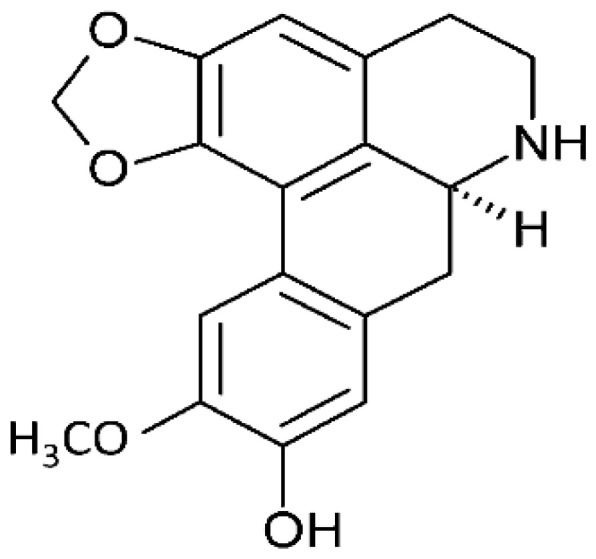	Induces apoptosis through ROS-mediated mechanisms. It targets the caspase apoptotic pathway through the activation of caspase 3, 7, and 9.	HHCM (Mahlavu hepatocellular carcinoma)	[25, 26]
Soybeans	Genistein	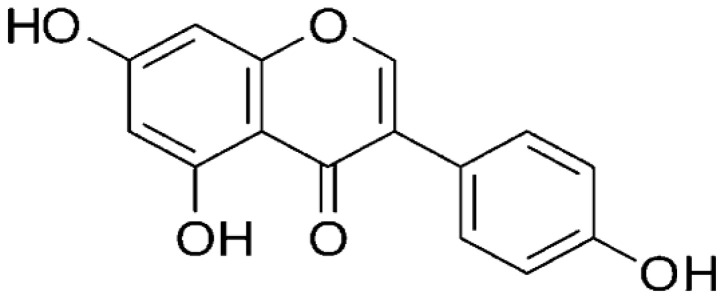	Targets the ATM/p53 dependent pathway and arrests tumor cells at the G_2_ and mitotic phase (M-phase) of the cell cycle.	Colon cancer	[27]
*Curcuma longa*	Curcumin	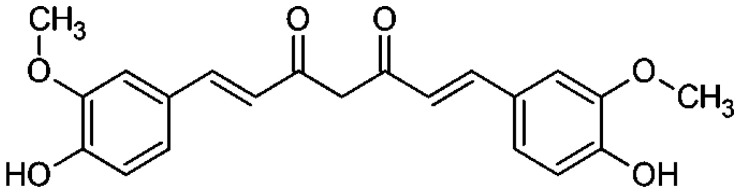	Inhibits cyclooxygenase (COX-2). Downregulates the activating protein-1, and β-catenin transcription factor, and upregulates the Fas-mediated apoptotic pathway.	Colorectal Cancer	[28]
Citrus fruits, green vegetables, onion, apples, and green tea	Quercetin	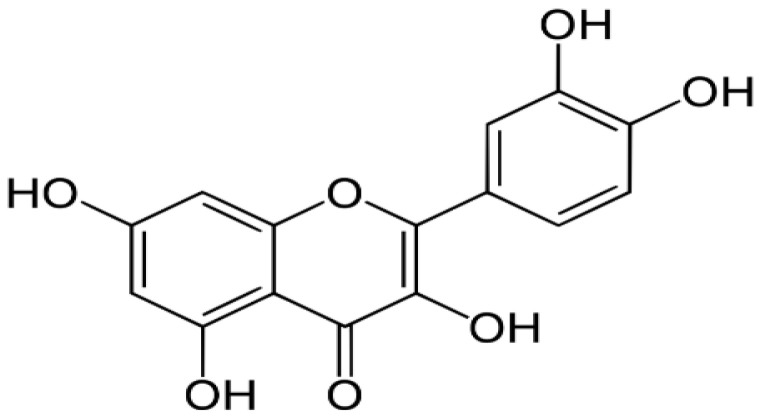	Targets and activates caspase 9 and induces apoptosis through the stimulated release of cytochrome c.	Breast, Colorectal, Lung, Kidney, Prostate, and Pancreatic cancers	[29–31]
Grapes	Resveratrol	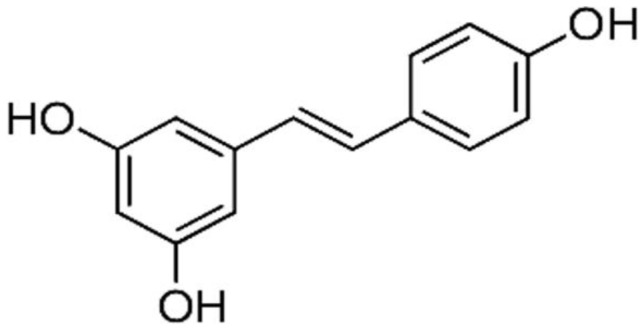	Targets the CD95 signaling pathway. It inhibits the expression of CDK 2, CDK 4, and CDK 6. It downregulates the expression of cyclin D (1 & 2) and cyclin E.	Breast, Colon, Liver, Pancreatic, and ovarian cancers	[31, 32]
*Scutellaria baicalensis*	Baicalein	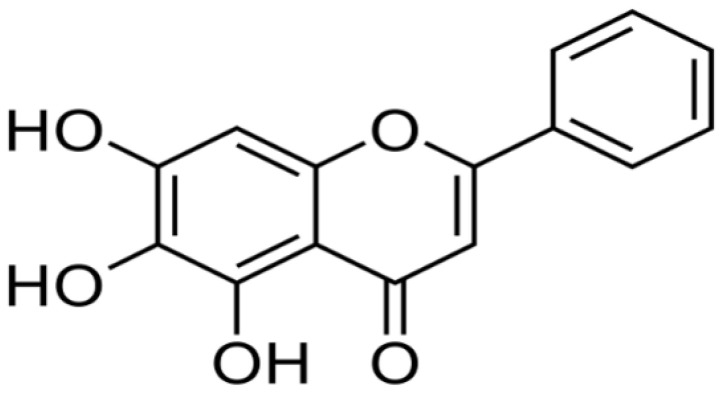	Inhibits the activity of mitogen-activated protein kinase (MARPK). It also inhibits the function of topoisomerase II.	Colon cancer	[2, 31]
*Glycyrrhiza glabra*	Licochalcone A	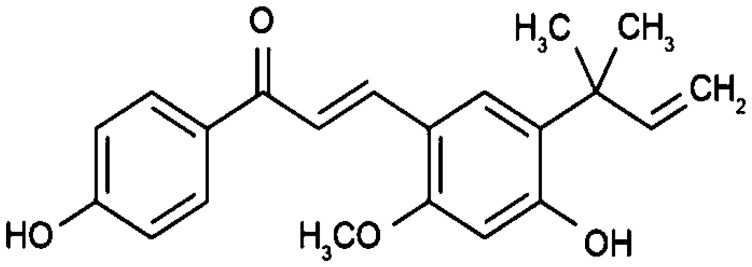	Arrests cell cycle at G_0,1,2_, or M-phase through its interactions with cyclin-dependent kinases (CDKs).	Brain cancer	[2, 33]

^*a*^major source of bioactive compounds used in the treatment of various cancers. ^*b*^plant-derived compounds used in the treatment of cancer. ^*c*^chemical structures of plant-derived anticancer compounds. ^*d*^mechanisms of action exhibited by plant-derived bioactive compounds in different cancers. ^*e*^cancer types treated using major plant-derived bioactive compounds.

**Figure 1:**
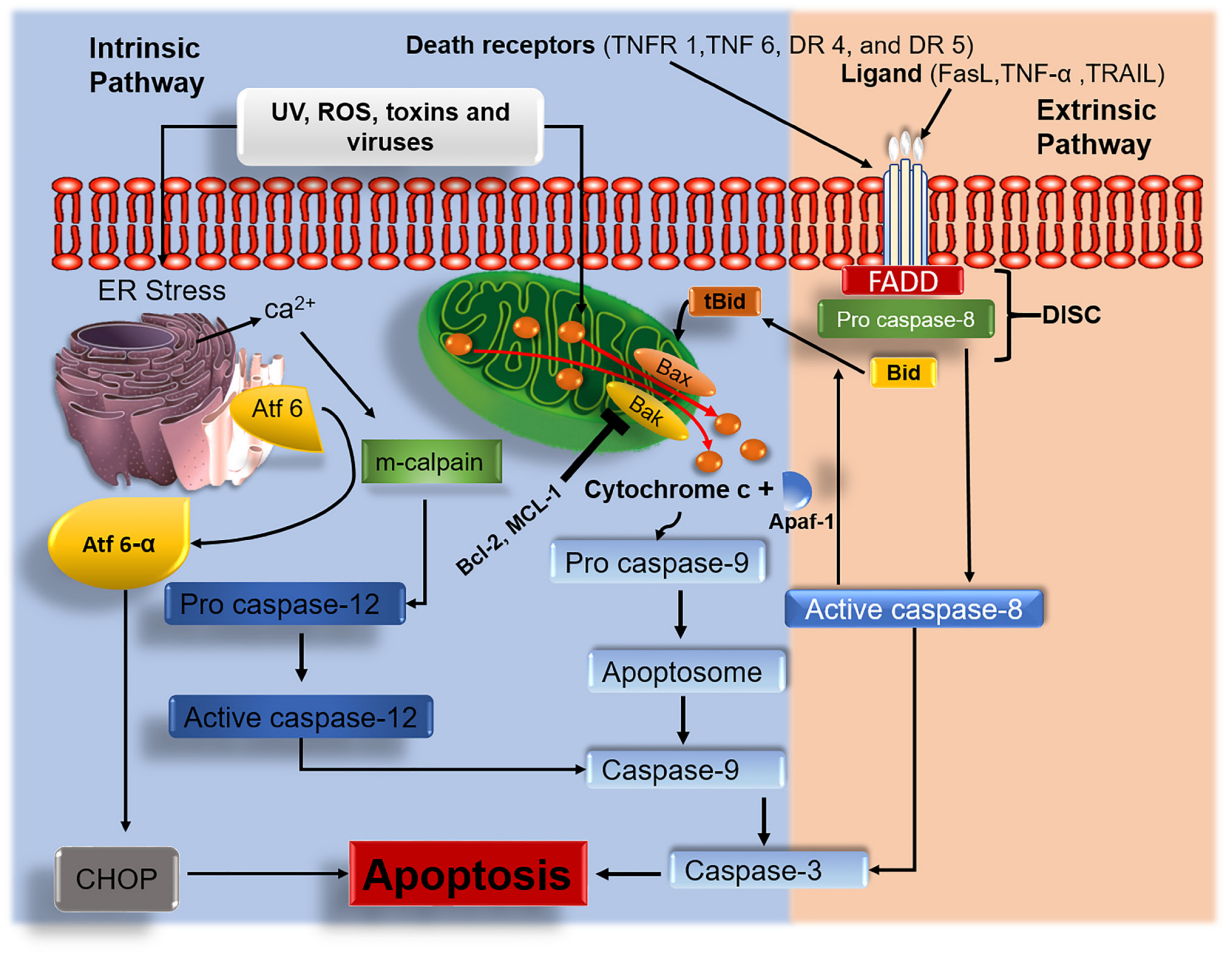
Intrinsic and extrinsic apoptotic signaling pathways. Anticancer agents target and interfere with the intrinsic and extrinsic signaling pathways. Upon treatment with tumor cells, anticancer agents induce ROS generation which eventually leads to damage of the mitochondrial membrane. Activated Bak/Bax results in mitochondrial permeabilization. Damaged mitochondrial membrane results in the release of cyt c, which triggers the activation of caspase-mediated apoptosis. Endoplasmic reticulum (ER) stress causes the discharge of calcium ions, which activates the m-calpain caspase-mediated apoptotic pathway. ER stress release activating transcription factor 6 alpha (Atf6-α) leading to apoptosis via activation of C/EBP homologous protein (CHOP). In the extrinsic pathway, the induction of death-inducing signaling complex (DISC) associated caspase 8 with the activation of apoptosis effector protein caspase 3 as well as BH3-only pro-apoptotic protein Bid to its active form tBid subsequently stimulates the multidomain pro-apoptotic proteins (Bax/Bak) to exhibit their activities.

**Figure 2:**
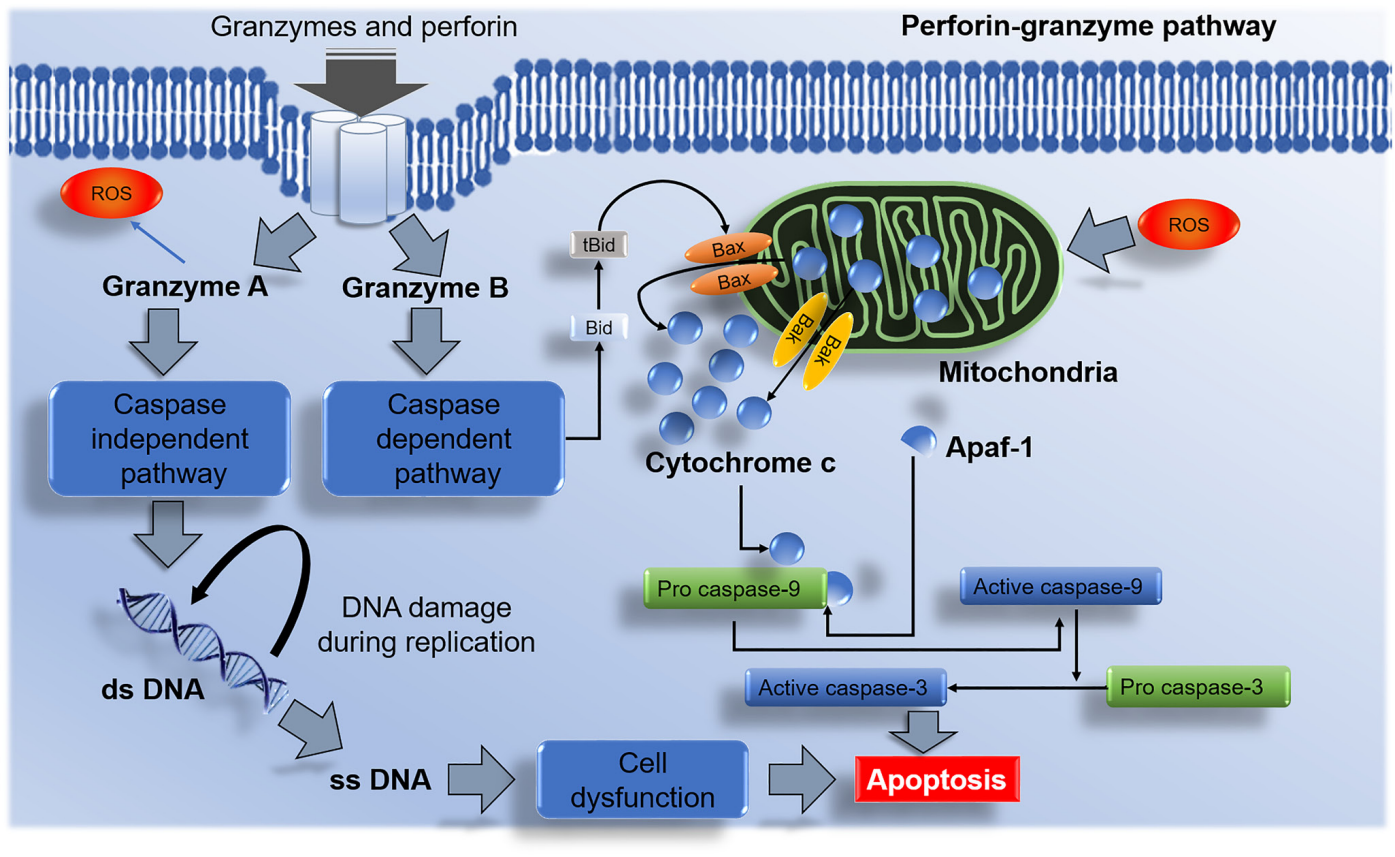
Perforin-granzyme NK cells mediated apoptotic pathway. Granzyme A activates the caspase-independent pathway and results in DNA damage and apoptosis. Granzyme B initiates the caspase-dependent pathway via Bax/Bak interactions with the mitochondria and releases cytochrome c. The discharge of cytochrome c from the mitochondrion leads to apoptosome formation with the aid from apoptotic activator protease factor 1 (Apaf-1) and pro caspase-9 which in the process gets activated and initiates the stimulation effector caspase-3 and induces apoptosis.

Cell cycle and apoptotic signaling pathways play an essential role in cell development. Under normal cell physiology, the rate at which cells divide should be equal to the rate at which cells undergo apoptosis. Two major pathways (intrinsic and extrinsic) are involved (Figure 1) to maintain this balance. Apart from these two major pathways, the third apoptotic pathway a perforin-granzyme pathway is also involved in homeostasis [25] (Figure 2).

## INTRINSIC PATHWAY

The mechanism of apoptosis in this pathway targets the mitochondria, and endoplasmic reticulum (ER) (Figure 1). Different molecules play a significant role in this apoptotic pathway. Reactive oxygen spices (ROS) are chemically reactive molecules containing oxygen. Increased generation of cellular ROS can lead to the damage of cell membrane, organelles, nucleic acids, proteins, lipids as well as activation of apoptotic signaling pathways [34].

BH3-only pro-apoptotic proteins are a subgroup of the Bcl-2 family of proteins. Members of these proteins include Bid, Bad, Bim, Puma, and Noxa. These pro-apoptotic proteins are capable of binding onto anti-apoptotic proteins (Bcl-B, Bfl-1, Bcl-2, and/or Mcl-1) and inhibit their anti-apoptotic activities [19, 35]. Other than inhibition, BH3-only pro-apoptotic proteins (Bid and Bim) may indirectly trigger the activation of the effector multidomain proteins Bax/Bak. Active forms of Bax and Bak oligomerize and lead to mitochondrial outer membrane permeabilization (MOMP) [36]. Once the mitochondrial outer membrane has undergone permeabilization, cytochrome c (cyt c) escapes the mitochondrion and enters the cytoplasmic matrix where it later begins to interact with apoptotic protease activating factor 1 (APAF 1) forming apoptosome which initiates the activation of caspase-9. Once caspase-9 is activated, it triggers the activation of pro caspase-3 and further leads to the execution of cellular apoptosis [37]. It is also noted that apoptosis can also be induced once the ER undergoes stress. As the ER undergoes stress, calcium ions are released into the cytoplasm, where they stimulate m-calpain to promote the activation of pro caspase-12 to its active form caspase-12. Activated caspase-12 on the other hand induces the activation of caspase-9, which eventually triggers the activation of effector caspase-3 which induces apoptosis. Another significant protein released by the ER as it undergoes stress is known as activating transcription factor 6 alpha (Atf 6-α). The release of this endoplasmic protein leads to the activation of C/EBP homologous protein, which eventually leads to apoptosis as shown in (Figure 1).

## EXTRINSIC PATHWAY

The extrinsic pathway is an apoptotic signaling pathway that is initiated by external signals to induce apoptosis. The first step in the initiation of this pathway involves death-ligands binding onto tumor necrosis factor (TNF) [14]. There are various types of death ligands, these include Fas ligand (Fas-L), tumor necrosis-related apoptosis-inducing ligand (TRAIL), and TNF [38]. The death receptors (DRs) play a vital role by mitigating the activation of the extrinsic pathway. Death receptors are a subunit of the tumor necrosis factor receptor superfamily [39]. These DRs include TNFR 1, TNF 6, DR 4, and DR 5 [40]. Fas-associated death domain (FADD) is another essential adaptor protein in the extrinsic pathway that is bound by initiator pro caspase-8 forming a complete death-inducing signaling complex (DISC). DISC is a multiprotein complex that is established by the interactions between DRs and death effectors of FADD and pro caspase-8 [41]. Pro caspase-8 is activated by the DISC and triggers the activation of pro caspase-3 to activated caspase-3 which will lead to cell death [23].

## PERFORIN-GRANZYME PATHWAY

The perforin-granzyme pathway is a cytotoxic lymphocyte-mediated apoptotic pathway that uses the Fas ligand (FasL) and perforin-granzyme to induce cellular apoptosis [42]. Perforin is a protein responsible for the formation and development of cell membrane pores. There are three main sources of perforin such as NK cells, CD4 positive, and CD8 positive T-cells [43]. Granzymes are a family of serine proteases that are contained within cytotoxic lymphocyte cells [44]. At least five granzymes such as A, B, H, K, and M have been discovered in humans [45].

Once granzymes are secreted by cytotoxic lymphocytes, they begin to enter the target cell. This is the first and essential step in cell death. In the perforin-granzyme pathway, granzyme A targets cellular DNA replication mechanisms which lead to the formation of single-stranded DNA nicks which eventually lead to cell dysfunction as illustrated in (Figure 2). It also induces the destruction of the mitochondrial membrane through the production of ROS which leads to mitochondrial dysfunction and eventually cells death [46]. In contrast to granzyme A, the function of granzyme B is to stimulate a rapid activation of the caspase-dependant apoptotic pathway leading to mitochondrial membrane destruction through cleavage of BH3-only pro-apoptotic protein (Bid). The interaction of Bid with pro-apoptotic Bcl-2 family proteins (Bax/Bak) result in the escape of mitochondrial mediators such as cyt c into the cytoplasm which later triggers the activation of the caspase dependant pathway [45].

## PRO-APOPTOTIC AND ANTI-APOPTOTIC PROTEINS

Pro-apoptotic and anti-apoptotic proteins are a group of proteins that belongs to the Bcl-2 family of proteins. Pro-apoptotic proteins promote apoptosis, and they are divided into two subgroups; multidomain pro-apoptotic proteins that consist of Bak, Bax, and Bok and the BH3-only pro-apoptotic proteins that consist of Bid, Bim, Bad, Noxa and, Puma. Anti-apoptotic proteins are proteins that inhibit apoptosis, and these consist of Mcl-1, Bfl-1, Bcl-X_L_, Bcl-2, Bcl-w, and Bcl-B [47].

There are various factors identified with a significant role in the cellular apoptotic process. The most important ones include the Bcl-2 family proteins, caspases (casp), and p53 [48, 49]. The fundamental role of these proteins includes the regulation, activation, or inactivation of the pro-apoptotic and anti-apoptotic factors [50]. Inhibition of pro-apoptotic multidomain Bax/Bak is the critical feature of oncogenesis. Similarly, the elevation of anti-apoptotic proteins promotes restrictions of apoptotic activities in tumor cells as well as the development of resistance to the immune system [35].

### Bcl-2 family of proteins

Bcl-2 family of proteins is a group of proteins that control cell death through direct interaction with the mitochondrial outer membrane. The Bcl-2 family of proteins is divided into three subgroups based on their function [50] as shown in Table 2. Their interactions with mitochondrial membrane may lead to mitochondrial outer membrane permeabilization (MOMP) and eventually induces the discharge of mitochondrial intermembrane proteins which include pro-apoptogenic factor cyt c into the cytoplasmic space. To initiate MOMP, there is supposed to be a balance between pro-and anti-apoptotic Bcl-2 family proteins in the cytoplasm [35]. The release of cyt c leads to the initiation of caspase-mediated apoptosis [51]. The role of anti-apoptotic proteins (Bcl-B, Bcl-2, Bfl-1, Bcl-w, Bcl-xL, and Mcl-1) is to avert cyt c release and preserve the integrity of the mitochondria while Bax, Bak, and Bok promote MOMP [35].

**Table 2:** Classification, role, and interaction of Bcl-2 family proteins in cancer

Bcl-2 family protein (Sub-family)^a^	Activity^b^	Role in cancer^c^	Bcl-2 family interacting proteins^d^	References
Bax (Multidomain)	Pro-apoptotic effector	Targets the endoplasmic reticulum, and the mitochondria. When activated, it promotes mitochondrial fusion in non-cancerous cells and fission in apoptotic cells.	Mcl-1, Bfl-1, Bcl-X_L_, Bcl-2, Bcl-w, Bcl-B, Puma, Bim, and tBid	[47, 55, 56]
Bak (Multidomain)	Pro-apoptotic effector	Binds to the mitochondrial outer membrane (MOM) and endoplasmic reticulum membrane (ERM). It promotes mitochondrial fusion in normal cells and fission in apoptotic cells.	Mcl-1, Bfl-1, Bcl-X_L_, Puma, Bim, and tBid	[47, 55, 57]
Bok (Multidomain)	Pro-apoptotic effector	Targets MOM and promotes MOMP. Its induction in apoptosis is independent of BH3-only activation.	Mcl-1 and Bfl-1	[55–58]
Bid (BH3-Only)	Pro-apoptotic initiator	Upon its activation by caspase 8, tBid gets confined within the location of MOM. It promotes apoptosis via the activation of apoptotic proteins (Bax/Bak) to permeabilize MOM.	Mcl-1, Bfl-1, Bcl-X_L_, Bcl-2, Bcl-w, Bcl-B, Bax, and Bak	[51, 55, 56]
Bim (BH3-Only	Pro-apoptotic initiator	Inhibits anti-apoptotic proteins. It directly binds and activates multidomain pro-apoptotic effectors.	Mcl-1, Bfl-1, Bcl-X_L_, Bcl-2, Bcl-w, Bcl-B, Bax, and Bak	[55, 59]
Bad (BH3-Only)	Pro-apoptotic initiator	Inhibits anti-apoptotic proteins which allow the activation of multidomain pro-apoptotic effectors.	Bcl-2, Bcl-X_L_ and, Bcl-w	[55, 59]
Noxa (BH3-Only)	Pro-apoptotic initiator	Inhibits anti-apoptotic proteins which allow the activation of multidomain pro-apoptotic effectors.	Mcl-1, and Bfl-1	[55, 59]
Puma (BH3-Only)	Pro-apoptotic initiator	Inhibits anti-apoptotic proteins. It directly binds and activates multidomain pro-apoptotic effectors.	Mcl-1, Bfl-1, Bcl-X_L_, Bcl-2, Bcl-w, Bax, and Bak	[55, 59]
Bcl-B	Anti-apoptotic factor	Selectively inhibits Bax pro-apoptotic effector and BH3-only pro-apoptotic initiators.	Bax, Bim and, tBid	[55, 57, 59]
Bcl-w	Anti-apoptotic factor	Inhibits Bax pro-apoptotic effector and BH3-only pro-apoptotic initiators.	Bax, Bad, Bim, tBid and, puma	[55, 57, 59]
Bcl-2	Anti-apoptotic factor	Inhibits both pro-apoptotic effectors and BH3-only pro-apoptotic initiators.	Bax, Bad, Bim, tBid and, Puma	[55, 57, 59]
Bcl-X_L_	Anti-apoptotic factor	Inhibits both pro-apoptotic effectors and BH3-only pro-apoptotic initiators.	Bax, Bak, Bad, Bim, tBid and, puma	[55, 57, 59]
Bfl-1	Anti-apoptotic factor	Inhibits both pro-apoptotic effectors and BH3-only pro-apoptotic initiators.	Bax, Bak, Bok, Bim, tBid, Noxa and, puma	[55, 57, 59]
Mcl-1	Anti-apoptotic factor	Inhibits both pro-apoptotic effectors and BH3-only pro-apoptotic initiators.	Bax, Bak, Bim, tBid, Noxa and, Puma	[55, 57, 59]

^a^member of the Bcl-2 family of proteins expressed in various types of cancer. ^b^apoptotic activities of members of proteins belonging to the Bcl-2 family of proteins. ^c^roles of various members of the Bcl-2 family of proteins in cancer. ^d^interactions of different members of the Bcl-2 family of proteins in cancer.

**Figure 3:**
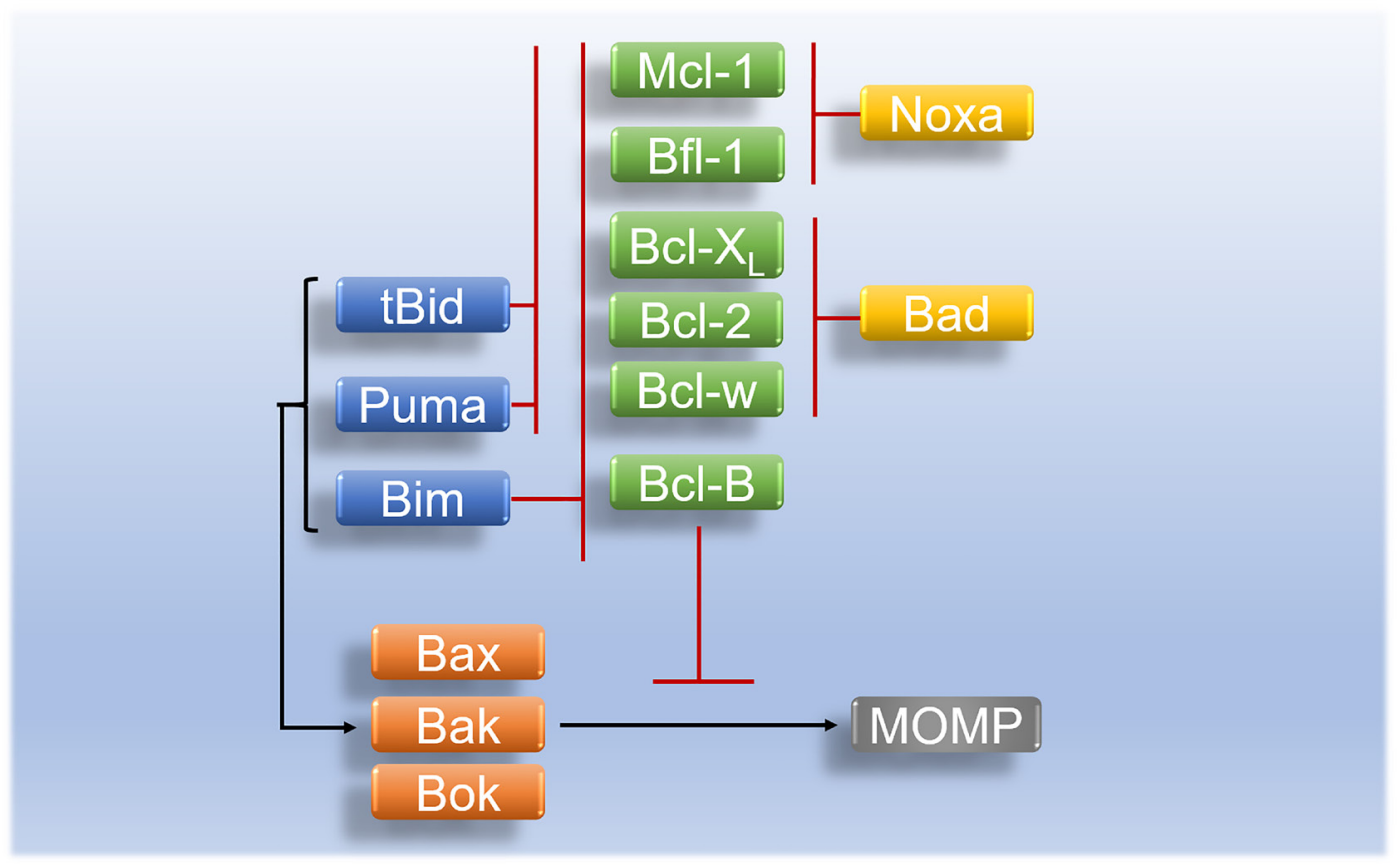
Regulation and interaction of Bcl-2 family proteins in apoptosis. Activated tBid and Puma inhibit anti-apoptotic proteins (Bcl-2, Bcl-w, Bfl-1, Bcl-X_L_, and Mcl-1) whereas activated pro-apoptotic initiator Bim inhibits anti-apoptotic proteins (Bcl-B, Bcl-2, Bcl-w, Bfl-1, Bcl-X_L_, and Mcl-1). Pro-apoptotic BH3-only protein Bad inhibits anti-apoptotic proteins of (Bcl-w, Bcl-X_L_, and Bcl-2) while Noxa binds preferentially onto anti-apoptotic proteins (Mcl-1, and Bfl-1) and inhibit their activity in apoptosis. Activated apoptotic initiators (Bim, tBid, and Puma) initiate the activation of pro-apoptotic effectors (Bax/Bak or Bok) which leads to MOMP. Pro-apoptotic effectors are inhibited by anti-apoptotic proteins (Mcl-1, Bfl-1, Bcl-X_L_, Bcl-2, Bcl-w, and Bcl-B).

Another subgroup of Bcl-2 family protein also known as single pro-apoptotic BH3-only proteins is comprised of pro-apoptotic proteins Bim, Bad, Bid, Puma, and Noxa [35]. The role of Bim and Bid in the tumor apoptotic pathway is to directly bind and induce the activation of Bak/Bax. Genetic interference with Bid and Bim may result in the modest decline of intrinsic apoptotic activities. Puma genes are essential in the regulation of cellular apoptosis. They are p53 upregulated apoptotic moderators, once activated they produce two transcripts; puma – α and β which activate Bax and Bak via the action of Bid and Bim [52]. Unlike other BH3-only pro-apoptotic proteins that can bind to all anti-apoptotic proteins, Noxa can bind to Bfl-1/A1, and Mcl-1 [53]. Just like other BH3-only family proteins, Bcl-2 associated agonist of cell death (Bad) triggers the initiation of tumour cell death by binding and neutralization Bcl-xL anti-apoptotic protein [54] (Figure 3).

### Caspases

Caspases belong to a family of proteolytic enzymes that play an important role in triggering the activation of apoptotic pathways. Based on their apoptotic function, caspases are subcategorized into initiator caspases which include caspase- 2, 8, 9, and 10. Examples of executioner caspases include caspase- 3, 6, and 7 [60]. Although the structure and function of caspase-2 are indistinguishable from other initiator caspases, its mechanism of action in apoptosis is less clear [61]. In the intrinsic pathway, recruitment of adaptor protein FADD the initiator of caspase-8 activations is initiated through their interactions with death ligand receptors. The activated form of caspase-8 activates the intrinsic pathway via the action of Bid. This whole process promotes the formation of MOMP and eventually, leakage of cytochrome c into the cytoplasmic space. The formation of apoptosome by the cyt c and Apaf-1 leads to the activation of caspase-9. The activated form of caspase-8 and caspase-9 can induce the activation of effector caspase that directly stimulates apoptosis caspase- 3, 6, and 7 [62].

### P53 gene

Tumor suppressor gene TP53 is a gene that codes for tumor suppressor protein 53 (p53), which plays a significant function in tumor cell death. The p53 protein is a transcriptional factor that regulates the transcription of target genes [63]. In most cases, p53 protein gets activated after DNA damage and later induces specific sequences that mediate apoptosis. Upon activation, p53 protein does not only prevent the transformation of tumor cells but also aims at eradicating cancerous cells [50]. In mammalian cells, p53 plays a significant role by regulating postmitotic checkpoints to prevent replication errors. Cell cycle arrests help the cells to improve their chances of survival by allowing possible repair time [64]. Gene expression studies conducted on mice showed that p53 upregulates the activation of p21 a cyclin-dependent kinase inhibitor (CDKI). However, the expression of p21 is mediated, and linked to the action of p53 [49]. PUMA is one of the critical members BH3-only family that induces apoptosis via the p53. It also induces the initiation of caspase cascade through modulation of Bax activities to aid the release of cyt c from mitochondria [65]. In normal cells, the level of p53 is regulated and maintained by mouse double minute 2 (MDM2) which binds to p53 and regulates its activity by facilitating its degradation [66].

## CELL CYCLE CHECKPOINTS AND ARREST

The cell cycle checkpoints are an essential machinery component of cellular surveillance that monitors the integrity and fidelity of significant proceedings of the cell cycle. It involves a chain of processes before the division of a parent cell into two progenies [67]. The cell cycle process consolidates five different phases which include G_(0, 1, 2)_, S, and M-phases. These phases are regulated by three checkpoints (G_0/1, 1,_ and _2_). Progression from one phase to another is regulated by CDK levels [68]. Cyclin-dependent kinases are regulatory enzymes that regulate proliferation via the regulation of distinct checkpoints of the cell cycle [69]. CDKs belong to a group of threonine-specific protein kinases. Their activation is dependent on the availability of cyclins [70].

**Figure 4:**
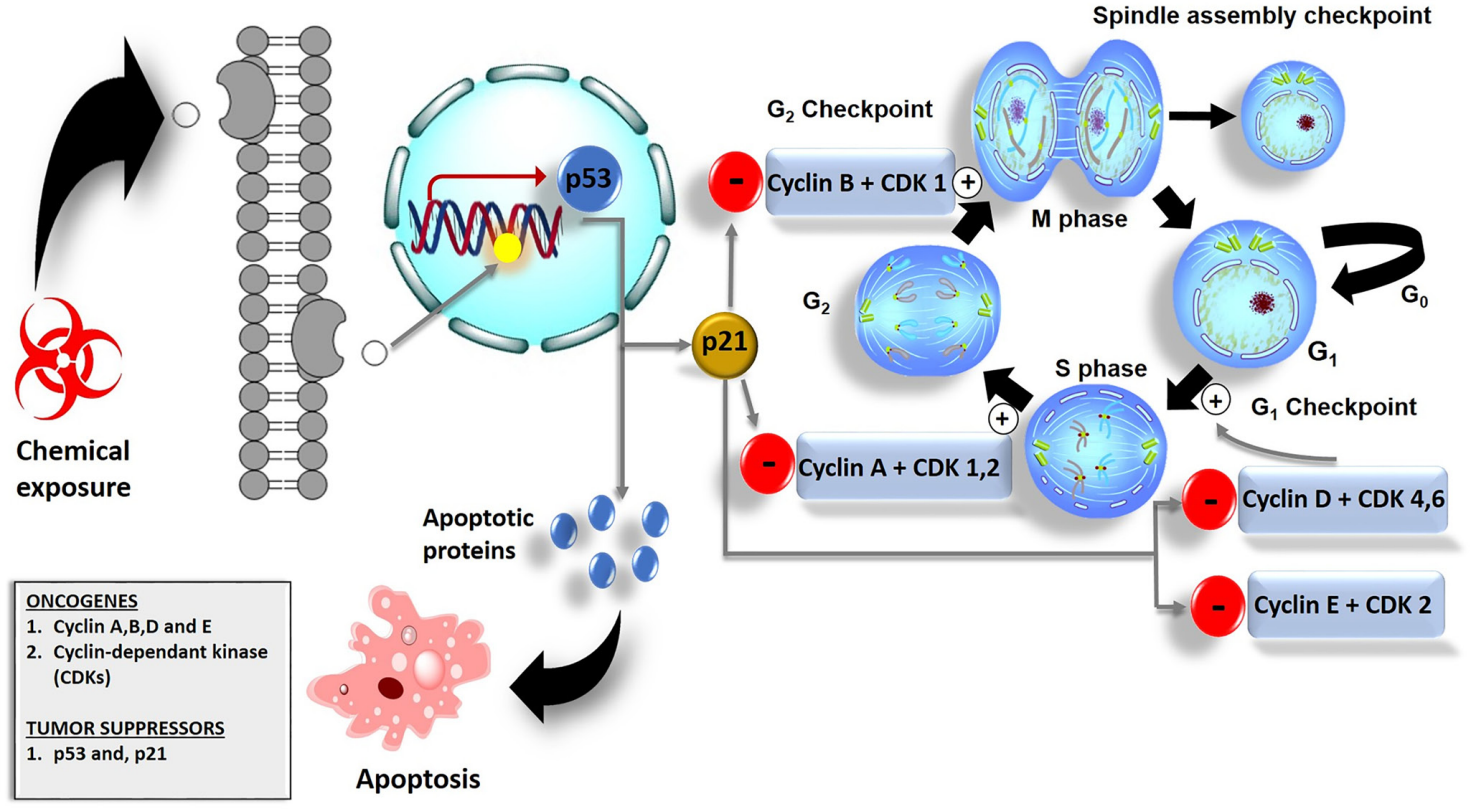
Cell cycle checkpoints and their role in cancer cell death. The G_1_ checkpoint checks for nutrients, growth factors, and the integrity of DNA. In the G_2_ phase, the G_2_ checkpoint checks for cell size. In case of DNA damage, the G_2_ checkpoint allows the damaged DNA to repair. Spindle cell cycle checkpoint checks for chromosomal attachment at the spindle i.e., before division. p53 initiates the expression of p21 proteins which down-regulates the activity of cyclins and CDKs and leads to cell cycle arrest.

The unregulated proliferation of cells may result from oncogene activation and inactivation of tumor suppressor genes such as the p53 gene. Mutation of these genes may result in overexpression of the genes which will allow the cells to bypass cellular checkpoints or may lead to cell cycle arrest [71] as shown in (Figure 4). However, cells do have a mechanism that prevents the progression of cells with damaged DNA from entering either the G_1_ or G_2_ phases of the cell cycle. In a case where DNA gets damaged, the levels of p53 in the cell increase. The p53 can act as a transcription factor in the regulation of cell growth [72]. It promotes the expression of p21 proteins and transcription induction of pro-apoptotic proteins such as the BH3-only proteins. To halt the cell from entering into different phases of the cycle, p21 binds onto cyclins and CDKs thereby inhibiting their oncogenic activities in G_1, 2,_ and S phases of the cell cycle [73] as shown in (Figure 4).

## CONCLUSION AND FUTURE PERSPECTIVES

In conclusion, Bcl-2 family proteins, caspases, and p53 play a significant role in apoptosis. Other than pro-apoptotic and anti-apoptotic proteins, it is more evident that other mitochondrial components such as cytochrome c contribute to the execution of the MOMP and cellular apoptosis. Another fascinating issue is that these proteins regulate intrinsic and extrinsic apoptosis. There are various ways through which tumor cell apoptosis is induced, they include intrinsic stress to the endoplasmic reticulum or mitochondria. Cellular DNA damage is also known for inducing the initiation process of apoptosis through a caspase-mediated apoptotic pathway. The significance of death receptors in tumor cell death is to induce the initiation of the extrinsic apoptotic pathway. The interactions of cell cycle regulators with apoptotic factors have a great impact on the fate of cell survival in multicellular organisms. The discovery of these proteins is helpful in the development of therapeutic approaches with improved selectivity and reduced side effects. Natural products may be a possible therapeutic agent alongside photodynamic therapy for the control and treatment of cancer. The use of nanoparticles is a promising therapeutic modality for the treatment of cancer. However, the use of plant-derived compounds and extracts in the synthesis of potent nanoparticles of medicinal application is one of the novel are of research that needs to be explored. This is because of the presence of diversified bioactive anticancer phytocompounds that are less toxic to humans, eco-friendly, and cost-effective.
